# ‘Students-as-partners’ scheme enhances postgraduate students’ employability skills while addressing gaps in bioinformatics education

**DOI:** 10.1080/23752696.2017.1339287

**Published:** 2017-06-21

**Authors:** Luciane V. Mello, Luke Tregilgas, Gwen Cowley, Anshul Gupta, Fatima Makki, Anjeet Jhutty, Achchuthan Shanmugasundram

**Affiliations:** ^a^ School of Life Sciences, University of Liverpool, Liverpool, UK; ^b^ Arthritis Research UK Centre for Integrated Research into Musculoskeletal Ageing (CIMA), Department of Musculoskeletal Biology, Institute of Ageing and Chronic Disease, University of Liverpool, Liverpool, UK; ^c^ Institute of Integrative Biology, University of Liverpool, Liverpool, UK; ^d^ Centre of Genomics Research, University of Liverpool, Liverpool, UK

**Keywords:** Bioinformatics, employability, peer-learning, postgraduate, students-as-partners

## Abstract

Teaching bioinformatics is a longstanding challenge for educators who need to demonstrate to students how skills developed in the classroom may be applied to real world research. This study employed an action research methodology which utilised student–staff partnership and peer-learning. It was centred on the experiences of peer-facilitators, students who had previously taken a postgraduate bioinformatics module, and had applied knowledge and skills gained from it to their own research. It aimed to demonstrate to peer-receivers, current students, how bioinformatics could be used in their own research while developing peer-facilitators’ teaching and mentoring skills. This student-centred approach was well received by the peer-receivers, who claimed to have gained improved understanding of bioinformatics and its relevance to research. Equally, peer-facilitators also developed a better understanding of the subject and appreciated that the activity was a rare and invaluable opportunity to develop their teaching and mentoring skills, enhancing their employability.

## Introduction

Bioinformatics is a STEM (Science, Technology, Engineering and Mathematics) discipline that continues to present specific pedagogical challenges, even though the first bioinformatics-related education paper was published 19 years ago (Altman, 1998). Current biological research, such as that driven by next generation sequencing and other high-throughput methods, generates large amounts of complex data. There is therefore an urgent need, from industry as well as from academia, for individuals skilled in bioinformatics to deal with and extract maximum value from this data. Since its infancy, the best methodologies for delivering bioinformatics training have been a matter of ongoing discussion, reviewed in Welch et al. (2014). Bioinformatics education and training present challenges from both student and lecturer perspectives (Badotti et al., 2013; Via et al., 2013). For postgraduate students in particular, one key problem results from the inevitable diversity in the students’ academic backgrounds. Pitching a lecture to suit the background of every student is a major difficulty.

Over 10 years of teaching bioinformatics, the first author of this study (LVM) has become increasingly aware that bioinformatics is a subject that is new for many postgraduate students. Previous studies of a postgraduate bioinformatics module taken by students at the University of Liverpool showed that there were dramatic differences in students’ prior knowledge in bioinformatics as they commenced their postgraduate learning (Mello, 2014, 2016). Also, students can be divided into two main categories: those who want a career in bioinformatics, and those who simply need to be able to select a few appropriate bioinformatics tools to deal with the data they obtain from their experimental postgraduate research projects. The research project is the most substantial piece of work that students complete at postgraduate level (Grant, Hackney, & Edgar, 2014). The research is carried out mainly independently and an increasing number of research projects require bioinformatics analyses (Via et al., 2013; Williams & Teal, 2017). However, the nature and extent of the bioinformatics involved is not necessarily apparent at the outset of the project. The module discussed herein, that runs in the first semester of year 1 of students’ postgraduate studies must therefore equip students with a broad overview of bioinformatics applications, both theoretical and applied aspects.

Previous questionnaire-based student feedback (Mello, 2014) identified a lack of individual support to students, together with a frequent inability of students to relate the workshop training tasks to their own subject-specific research projects. The study presented here involved an intervention based on staff-student partnership. This entails students and lecturers working together, taking shared responsibility for the enhancement of student learning (Healey, 2012). This was coupled with peer-learning, which refers to the use of teaching and learning strategies in which students learn with and from each other without the immediate intervention of a teacher (Boud, Cohen, & Sampson, 2006). The student–student interaction involved senior PhD students acting as peer-facilitators to junior students from Master and PhD degrees (the peer-receivers). The main role of peer-facilitators in this study was to ensure that peer-receivers understand how these bioinformatics skills can be applied in real research settings and engage students with the module, rather than to assist lecturers in teaching theoretical concepts to peer-receivers. However, peer-facilitators also assisted lecturers as demonstrators in the practical workshops to guide the computer-aided practical sessions, which ensured further one-to-one or small group discussions between the two peer groups on the applicability of bioinformatics skills. In carrying out this role, it was also expected that the peer-facilitators would develop their transferable skills and thereby improve their employability.

### Peer-learning

Collaboration is essential in the scientific environment and discussions are enriched when understanding is shared, even if the benefits are weighted in favour of one party. In line with scientific teaching (Handelsman, Miller, & Pfund, 2007), students should be able to interconnect diverse subjects and choose appropriate tools to solve problems, especially at postgraduate level. Peer-learning has long been used in several STEM disciplines, where it has been shown to enrich students’ learning experiences while developing collaborative work skills (Burgess, McGregor, & Mellis, 2014). Peer-facilitation approaches have also been shown to increase student engagement, improve retention rates and attendance rates, and have led to better performance in exams (Congos & Schoeps, 2003; Drane, Micari, & Light, 2014; Stone & Jacobs, 2008; Power & Dunphy, 2010; Zhao & Kuh, 2004). Drane et al. (2014) reported on the effectiveness of peer-led learning over 10 years in a US research-led university across five STEM disciplines, showing improved retention rates and enhanced performance of undergraduate students. Although most of the early studies focus on the benefits of peer-learning approaches to students, several recent studies and reviews have illustrated the benefits to those delivering the peer-learning sessions. These benefits include opportunities to develop and practice teaching, assessment and communication skills; a gain of confidence through reflection and expansion of their own knowledge; an increased understanding of the subject and appreciation of educational theory and practice; enhanced confidence, leadership, teamwork, time management and evaluation skills; and improved personal adequacy and satisfaction (Burgess et al., 2014; Couchman, 2009; Latino & Unite, 2012; Park, 2002). When developing teaching skills, Han (2016) showed that a reflective approach is required from a teacher to properly evaluate her/his own teaching experiences. In addition, the author suggested that educators need to share their experiences to further develop themselves as teachers.

### Employability skills

Employability skills, also known by transferable skills, are defined by Knight and Yorke (2004) as ‘a set of achievements, understandings and personal-attributes that make individuals more likely to gain employment and be successful in their chosen occupations’. Therefore, while employment should be regarded as an outcome, employability should be viewed as a lifelong process (Pegg, Waldock, Hendy-Isaac, & Lawton, 2012).

The UK Engagement Survey analysed the development of students’ skills and abilities, linking them to skills sought by employers in graduates (Buckley, 2015). The analysis indicated that students who participated in extra-curricular or co-curricular activities during their studies were more positive about their skills development than those who had not engaged with them. The recently produced ‘Higher Education Academy’s Employability Information Pack’ (Norton, 2016) aims to help and support academics to review their institutional employability policy in order to produce highly skilled graduates while maintaining the provision of high-quality teaching and research. In 2011, 22% of all degrees awarded in the UK were in STEM disciplines (Katsomitros, 2013), and the number of students studying STEM subjects in the UK is at a record high according to the Higher Education Funding Council (Thomas, 2014). Prinsley and Baranyai (2015) reported that students with STEM qualifications are valued in the workplace, even when their major field of study is not a prerequisite for their role. Nevertheless, Zhou and Nhlanhla (2015) claim that Maths graduates, because they studied a science subject, lack communication skills, affecting their employability. The Wakeham Review of STEM Degree Provision and Graduate Employability showed a poor employment rate of Biological Sciences graduates, highlighting concerns regarding their skill sets, including their poor capacity for team work (Wakeham, 2016). At postgraduate level, the provision of further training skills for PhD students involved in STEM subjects is a requirement of the Research Councils (RCUK Impact Report, 2013). Abbas and Spacey (2015) discuss the sense of being employable from the student’s and academic’s perspectives, and show that these may differ. Taken together, all these studies indicate that more has to be done to engage students with activities to further develop their transferable skills. Furthermore, they must be helped to recognise transferable skills as they are acquiring them, and to relate them to their future employability.

### Study aims and research question

This study presents a teaching intervention on a bioinformatics postgraduate module taken by Master and PhD students at a UK university. The research question for this study was: ‘To what extent do student-staff partnership and peer-learning opportunities contribute to student learning and employability?’. The intervention involved students in peer-facilitator or peer-receiver roles. Peer-facilitators were PhD students who had previously taken the module and peer-receivers were current students enrolled in the module.

The intervention had several aims. For both peer-receivers and peer-facilitators, it was hoped that the intervention would improve their overall understanding of the application of bioinformatics in diverse areas of current biological research. In addition, for the peer-receivers, a second important aim was to foster students’ awareness of the relevance and transferability of the bioinformatics knowledge gained in the module to their own research projects. Improved module engagement and a better understanding of the module content were further objectives. For the peer-facilitators, the key aim was an enhancement of their employability skills. For them, the experience can be considered to be a valuable part of their doctoral training in light of the focus of UK research councils on developing employability skills, including teaching, among doctoral students (RCUK Impact Report, 2013).

## Methodology

A common research strategy used by teachers in their institutions to transform their teaching practice is action research (Cohen, Manion, & Morrison, 2007). Thus, the research design used here was action research, and in line with the principles of action research, feedback from a previous study (Mello, 2014) was used to guide the planning and execution of this study. Ethics permission was sought from and granted by the University’s ethics committee; and all students agreed to be part of this research study.

### The module and its intervention

The module, *Informatics for Life Sciences*, was designed based on the expertise of six members of staff teaching in the module to provide an overview of the use of bioinformatics in the biological sciences. It covered a broad range of topics: database searching, sequence alignment, genomics, phylogeny and evolution, protein modelling, population ecology and modelling metabolic pathways. The module was taken by students from different postgraduate degrees including those on Master (MSc, Integrated Master (MBiolSci) and MRes) and PhD programmes. Data derived from students enrolled on the module between 2013 and 2015 were used to test whether the aims of this study were achieved. The cohort sizes for the 2013–2014 and 2014–2015 academic years were 43 and 45, respectively.

The module comprised lectures and accompanying workshops, one of each per week for a total of 12 weeks. One hour lectures were delivered by a single lecturer, where the theoretical basis behind each bioinformatics topic was taught. These lectures were each supplemented by a two hour computer-based practical workshop, where students follow a pre-defined task, whilst being given the periodic class-wide guidance of a single lecturer. The face-to-face element was complemented by online resources/activities to support a self-directed learning approach by students (Mello, 2016).

The intervention changed the module by introducing short talks from the peer-facilitators in preparation for the workshops. Peer-facilitators briefly presented their research project explaining how bioinformatics analysis was applied to their biological research questions. A direct connection to the bioinformatics topic of the lecture and workshop was made in each session (more detail below). Although not all peer-facilitators’ work has been published yet, examples of their publications are given in Table 1. The intervention aimed to determine if the provision of peer-learning would help peer-receivers understand the applicability of bioinformatics tools in their research projects, and enhance their engagement with the module while broadening their general appreciation of bioinformatics in the biological sciences. A second aspect to be examined was if the provision of a staff-student partnership associated with peer-learning would provide peer-facilitators with the opportunity to practise their teaching skills while improving their employability skills in general. The resulting data was derived from questionnaire-based surveys and from analysis of reflective logs. The activities and data collection are described in detail in the next sections.

**Table 1.** Bioinformatics topics covered in the module *Informatics for Life Sciences*, and peer-facilitators’ published work used in the workshop talks.

Bioinformatics topic	Peer-facilitator’s published work
Database searching	Shanmugasundram, Gonzalez-Galarza, Wastling, Vasieva, and Jones (2013, 2014)
Sequence alignment	Bogomolovas et al. (2014)
Genomics	Sibthorp et al. (2013); Wright, Makki, Moots, and Edwards (2017)
Phylogeny and evolution	Pounder et al. (2015)
Protein modelling	Bogomolovas et al. (2014)
Population ecology	Withenshaw, Devevey, Pedersen, and Fenton (2016)
Modelling metabolic pathways	Shanmugasundram et al. (2013, 2014)

### Peer-facilitator recruitment and involvement with the intervention

For the first year of activity, academic year 2013–2014, a preliminary invitation was disseminated, via e-mail, to 14 postgraduates from student cohorts that had completed the module in previous years. These postgraduates were selected by the course convenor based on their academic performance or their level of engagement whilst attending the module. In an initial meeting, students were briefed about their involvement in the study. The first phase involved the planning and delivery of the intervention. The second phase involved the dissemination of the work via conferences and article publication. Ten students agreed to participate in phase one, and six agreed to take part in both phases. In the 2014–2015 academic year, a peer-receiver from the 2013–2014 cohort acted as peer-facilitator in addition to the 10 peer-facilitators from the previous year. Thus, there were 10 peer-facilitators in the 2013–2014 session and 11 the following year. Peer-facilitators received training in oral presentations during the first year of their PhD. This training was in addition to compulsory demonstrator training received from the University of Liverpool.

Peer-facilitators’ research, including bioinformatics techniques currently being used in their research projects, was discussed with the course convenor during the initial intervention discussion sessions. Once matches between peer-facilitators’ expertise and the computer-based practical workshops topics were identified, two peer-facilitators were assigned to give a short presentation prior each workshop. Most peer-facilitators carried out this role for two workshops but some did three. In total, there were 12 workshops and 24 pre-workshop talks given by 10 (2013–2014) or 11 (2014–2015) peer-facilitators. The talk consisted of a brief overview of the facilitator’s project and their research question, together with why and how they used a particular bioinformatics technique to assist them in their research. These presentations were followed by a workshop on the techniques presented. All peer-facilitators were invited to attend the presentations of their peers.

Following the presentations, the peer-facilitators offered the peer-receivers a chance for further interaction and discussion. Interaction between peer-receivers and peer-facilitators was also encouraged during these workshops to obtain guidance on the workshop material. With respect to the aim of increasing the ability of students to relate the work to a real-life research setting, discussions on bioinformatics skills that would build on the workshop materials and, in particular, have the potential to be used in their own research projects, were encouraged.

### Data collection and analysis

Punch (2005) describes empirical research as research based on direct experience or observation of the world involving two main kinds of data, quantitative or qualitative. As the learning outcome from the activity differed for the two groups of students, peer-facilitators and receivers, two different questionnaires collecting quantitative and qualitative data were designed. In both cases, the questionnaire measuring technique used was Likert scale, yes/no and value response format (Allen & Seaman, 2007). Qualitative data was analysed using a thematic approach (Boud et al., 2006). Students’ participation in the survey was voluntary and anonymous.

#### Peer-receivers

For the peer-receivers, the aspects analysed in the survey were engagement with the module, understanding of bioinformatics, both broadly and as specifically relevant to their own project, and appreciation of the peer-facilitators’ participation. Questionnaires were handed out to peer-receivers during the final module workshop in the first year of study but distributed electronically for the second year.

#### Peer-facilitators

For this group of students, the survey emphasised development of their teaching and other key employability skills; and improvement of their understanding of bioinformatics within their own and other projects. The questionnaire was distributed electronically.

In addition to the questionnaire, qualitative data was also collected from peer-facilitators’ reflective logs. After their participation in the workshops, students were asked to record their personal impressions of the activity, including specific enquiries from peer-receivers. This was intended to enhance their reflexive learning (Han, 2016), and provide guidance for peer-facilitators involved in subsequent workshops when preparing and delivering their own presentations. In addition, peer-facilitators were asked to record their own reflections after the activity was complete. Although the identities of the peer-facilitators reflecting in the logbook were known, the peer-facilitators did not disclose the identities of individual peer-receivers.

Peer-facilitators’ reflective logs were initially read by the module organiser and by two of the peer-facilitators. They then met as a group to develop and identify emerging themes to guide data analysis. Three themes were derived from the reflective logs guidelines/questions, which were aligned with the research questions (Tables 2 and 3): personal satisfaction and motivation with the planning of the work and dissemination of the results; understanding of bioinformatics; and teaching and transferable skills. Analysis of these themes was divided according to the two project phases: (1) planning and during the project, and (2) retrospective reflection. During this stage, the trustworthiness of the study was addressed through member checking and data triangulation. To ensure data accuracy, we used individual member checking particularly regarding the selected quotes for each theme (Corbin & Strauss, 1990). No negative comment was reported by any peer-facilitator, and a selection of the positive comments is presented in the result section.

**Table 2.** Peer-facilitators statements during the activity planning.

*A. First thoughts about the project*
As an ecologist, it wasn’t immediately obvious that I needed bioinformatics skills, and so my engagement with this module was not wholehearted. In reality, however, I regularly use several tools in my research. I would therefore relish the opportunity to engage with students currently taking this course, and show them exactly how I have unexpectedly put bioinformatics theory into practice during my research
An additional strength of the project is the availability of experience in the form of previous students, using techniques that would have been of great benefit to us, in the same situation
Had such an opportunity been made available to me during my time studying the module, I believe that it would have made a great deal of improvement to both my learning and my research as a result
*B. Career development*
There are several reasons I was motivated to engage in the project. Firstly, as PhD students we are encouraged to engage in some form of teaching to strengthen their CV and this is a good and rare opportunity! Secondly, I think the experience of presenting and teaching in a different environment will prove invaluable to the development of my career
*C. Bioinformatics skills development*
I believe that retrospectively presenting my work in terms of the application of principles from this module will strengthen my own knowledge of my project and I wish I had the forethought to think how this module could enhance my own project while undertaking the module

**Table 3.** Peer-facilitators reflections in retrospect of the study.

*A. Personal satisfaction*
I enjoyed the satisfaction of having shared my personal research journey with others
Some students downloaded my slides from the VLE (students’ presentation)
I was really amazed by the level of response and active discussion in the workshop
I feel that tallying the outcomes of the project to achieve a publication has allowed some reflection on our achievements
I felt I was valued by the other senior academics when presenting at conferences as the presentation sparked questions and discussions aimed to peer-facilitators.
*B. Bioinformatics/research skills development*
I was able to learn more about bioinformatics applications from others peer-facilitators
Thinking precisely about how I used bioinformatics as part of my work gave me opportunity to view my own research in a new way
A pair of students asked for advice on analysing their data, having taken notes of similar analysis explained in the presentations
*C. Teaching*
It was really a rare and valuable opportunity to place myself between being a student and a tutor.
I was also able to learn a little about student behaviour. Good teaching experience
*D. Employability*
Although the interview for my current post-doctoral position was focussed on my genomics knowledge and research experience, one could argue that my peer-facilitator experience may have helped me reach wider audience from different biological backgrounds during interview seminar and discussions
Sharing the stage with senior academic in presenting this project has helped increase my confidence and time management skills in joint presentations
My proposal for teaching activities at the interview was built upon the experiences and opportunities to interact with experienced members of staff during this project. I would not have had an opportunity to further discuss teaching approaches prior to interview, if I didn’t participate in this collaboration expanding my network
I now work in a small company where effective teamwork is often necessary. I believe that describing previous experience of teamwork, like the students-as-partners project, during my interview highlighted my suitability for the role. My job also includes explaining complex subject matter in simple terms, for which this project provided plenty of experience
As a postdoc, I was an assistant instructor in the Wellcome Trust Advanced Course on Fungal Pathogen Genomics held at the Hinxtion Genome Campus. Here I demonstrated the database tools to participants (from PhD students to assistant professors from worldwide) and had discussion on how these tools could be used in their own research or what additional tools could be used to fulfil their own needs

## Results

### Peer-facilitator engagement in the planning process

Peer-facilitators received the idea of student–staff collaboration well and their opinion was taken into account during the design of the project. These students suggested the need for including strategies to help develop awareness of applications of bioinformatics in future cohorts of students, as they found this aspect to be missing when they undertook the module. This was evident from the statements from peer-facilitators shown in Table 2, *A*. The students from previous cohorts agreed to act as peer-facilitators citing this opportunity as a valuable and rare teaching experience for them. It was encouraging to note that they saw this proposal as mutually beneficial and were able to link such experiences to better career development (Table 2, *B*). It was also evident that the peer-facilitators believed that participation improved their understanding of bioinformatics concepts and applications, as early as during the planning stage (Table 2, *C*).

### Peer-receiver responses

**Figure 1.**
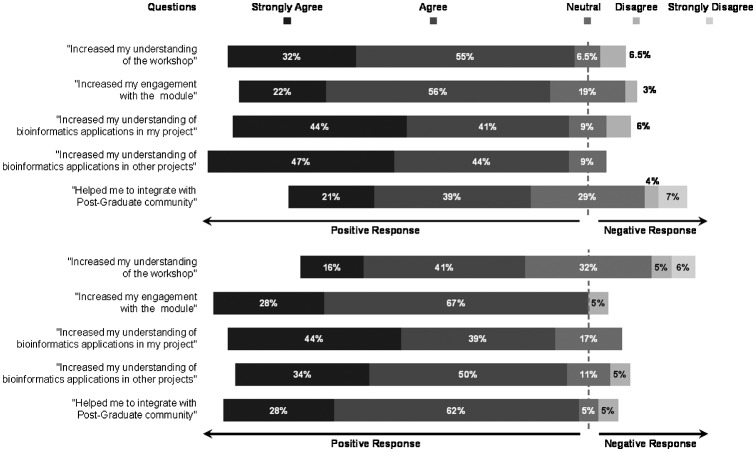
Questionnaire responses from peer-receivers over the two years of study (Top – 2013/2014 academic year; Bottom – 2014/2015 academic year) – diverging from the proportions of neutral responses outward to the proportions of positive and negative responses.

Notes: The trend shows a strong positive response towards the questions asked of the peer-receivers. Response level is represented as a percentage of questionnaire responders.

75 and 51% of the peer-receivers voluntarily filled the quantitative questionnaire-based survey in the two years of study, respectively, a total of 62%. About 78% of the peer-receivers agreed or strongly agreed that they engaged with the module and 87% of the respondents felt that the peer-facilitators helped them understand the subject content. A majority of peer-receivers also agreed that the peer-facilitation improved their understanding of bioinformatics applications in their own research projects (84% of respondents) and in others’ research projects (91% of respondents). 28 out of 32 peer-receivers responded to the question ‘whether the peer-facilitator engagement helped them integrate into the biology postgraduate society?’, of which 68% respondents agreed that it had facilitated their integration into the Research Institute’s Postgraduate Society at least to some extent (Figure 1). The student-run Society is for all Postgraduates in the Institute of Integrative Biology, and aims to support students through formal and informal activities.

### Peer-facilitator responses

**Figure 2.**
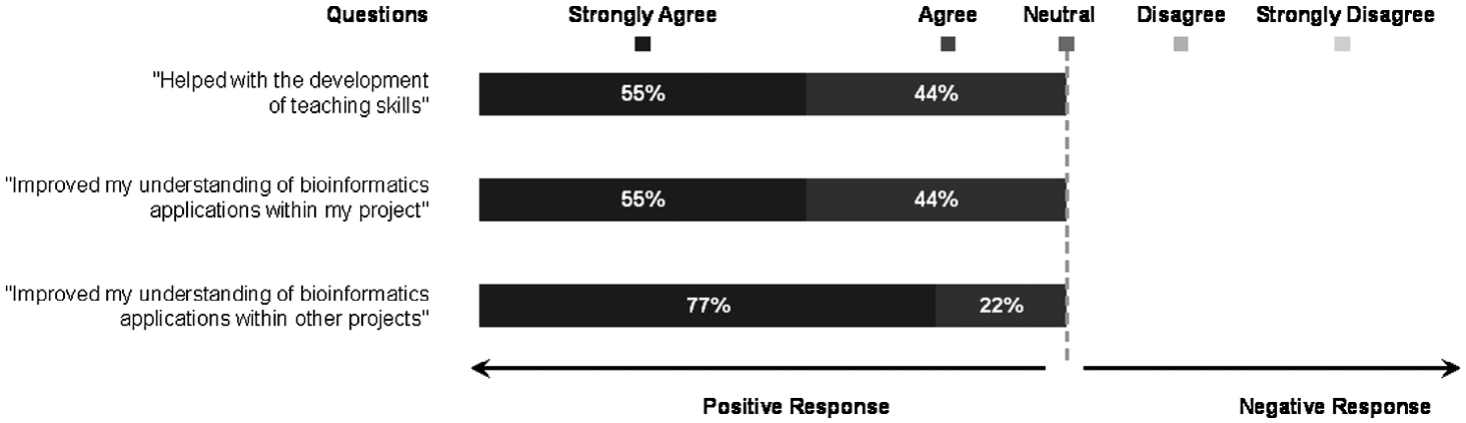
Questionnaire responses from peer-facilitators over the two years of study.

Notes: There is a strong positive response towards the aims of the study. The response-level is shown as a percentage of questionnaire responders.

All peer-facilitators (both years of study) either agreed strongly or agreed to some extent that their role as peer-facilitators has helped them develop their teaching skills (Figure 2). This was also evident from several comments that were made in the reflective logbook after their teaching session, and a few of those comments are presented in Table 3, *A*. Moreover, it was evident that presenting the experience of their research journey to their peers, as well as the experience of presenting the results of this study at Educational conferences and producing this publication, enhanced their level of personal satisfaction (Table 3, *B*). 100% of the respondents also agreed that their role as a peer-facilitator had assisted them in understanding the application of bioinformatics to their own and to their peers’ research projects (Figure 2). The reflections of peer-facilitators from the logbook also provided an insight into the involvement of peer-receivers in this new teaching strategy and expanded upon the quantitative results obtained from the questionnaire responses. This demonstrated that the peer-receivers recognised the importance of the contribution of peer-facilitators and appreciated that understanding the application of bioinformatics techniques could potentially provide them with solutions in their own research. It is highly likely that this will reinforce peer-facilitators’ confidence in the value of their own research and their contribution in peer-facilitation (Table 3, *C*). While some peer-facilitators are still working towards their PhD, six of them have completed it and have been successful with job applications. Examples of jobs: Research Associate, Research Technician, Patent Attorney and trainee on the NHS Specialist Training Programme in Health Informatics. The students claimed that their experience in this project has helped them present themselves and speak confidently to interviewers (Table 3, *D*). These statements clearly illustrate the mutual benefits that the peer-receivers and peer-facilitators derived from this peer-facilitation process. Importantly, the experience helped prepare students for different types of job.

## Discussion and conclusion

Rarely in current higher education system are opportunities available for extended interaction between staff and students: methods of communication promoting simple document transmission over staff–student dialogue generally predominate (Freeman, Millar, Brand, & Chapman, 2014). One solution highlighted by Freeman and collaborators’ study is the development of a partnership between students and academic staff aimed towards improved learning and teaching. The current study provided similar enhanced communication between staff and students in order to directly influence learning while enhancing and broadening postgraduate training; and developing employability skills. Involvement of postgraduate student facilitators in the development of teaching strategies, planning and knowledge dissemination provided benefits for all three academic tiers (staff, peer-facilitators and peer-receivers) involved. Techniques used to promote students as academic partners, also mentioned in Freeman et al. (2014), placed an emphasis on student-led project development. In the same way, the current study encouraged enhanced ownership of education development, and provided an opportunity to make a difference to student peers within the Faculty. This was reflected in feedback from both peer-receivers and peer-facilitators.

In this bioinformatics module, complete instruction of the subject by experienced academic staff, allied to the availability of peer-facilitators to demonstrate how a particular concept/tool was applied in their own research, was a combination that proved to enrich the student experience and improve understanding of the subject matter. The main focus of peer-facilitation in this context was neither demonstration nor small group teaching, rather a focus on discussion of the application of conceptual knowledge and techniques in real research projects. It is important to note that the respective peer-facilitators were the owners of the knowledge that they discussed with the peer-receivers and therefore took leadership in the activity. The peer-facilitators were supervised by the responsible academic staff during the workshops and therefore there was an opportunity for questions that could not be answered by the peer-facilitators to be directed to the academic staff and the answers explained to the student cohort. This also provided the peer-facilitators with an opportunity to fill any gaps in their own understanding of the subject area. In addition, peer-facilitators exercised their time management and communication skills while developing and delivering their presentation respectively. Importantly, some of the peer-facilitators engaged in the dissemination of this work at teaching conferences and are the co-authors of this research article. These opportunities challenged PhD students to think about and discuss pedagogical approaches, an opportunity rarely offered during postgraduate training. It was also evident from their comments that they felt valued by their peers and the Faculty. These benefits are consistent with the benefits that are reported from other comparable studies from different STEM disciplines (Burgess et al., 2014; Couchman, 2009; Curtis, Goodson, McDonnell, Shields, & Wyness, 2012; Latino & Unite, 2012; Park, 2002).

**Figure 3.**
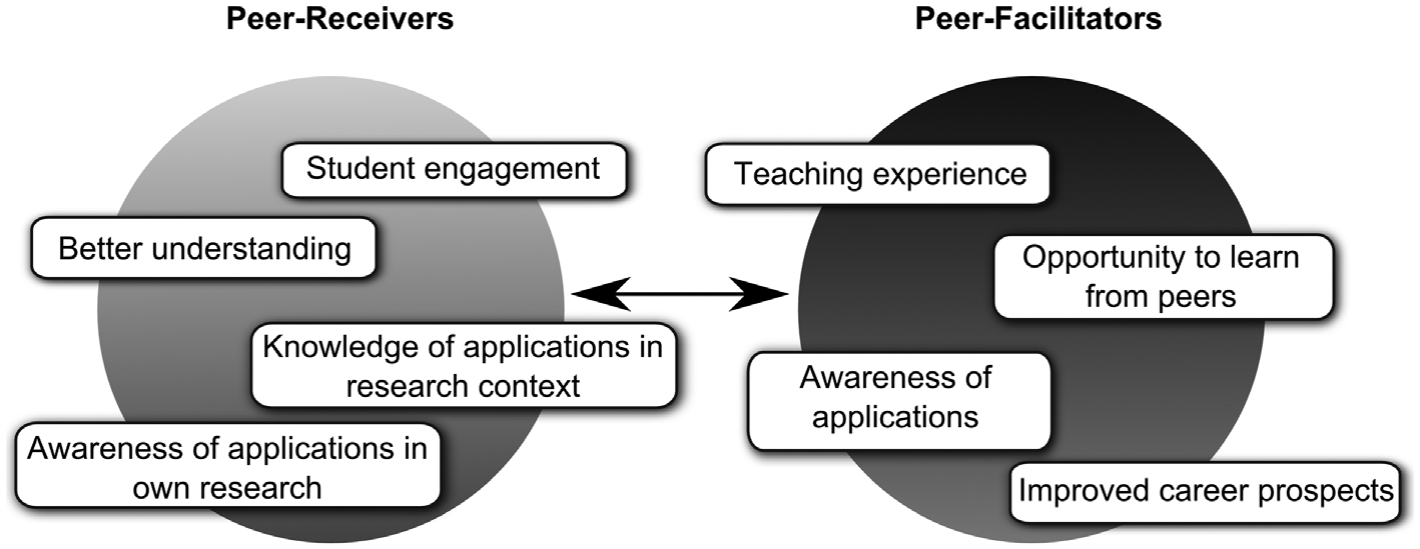
Diagrammatic representation of the bidirectional manner in which the benefits from the current study were perceived in the two years of study.

The current study provides an additional intermediary between student and lecturer in the form of experienced students, fostering a similar dynamic as achieved by Curtis et al. (2012), but involving greater facilitated learning through a more easily adopted horizontal association between past students and current students. In this way, each party could act as ‘co-producers of knowledge’, which would facilitate a dialogic learning environment. This can be compared to one of the outcomes from Curtis et al. (2012), which identifies the significance of reducing the mentality of a ‘novice’ and ‘expert’ relationship towards enhanced learning. Benefits to the peer-receivers came in the broadening of cross-disciplinary subject knowledge, with topics placed in the context of a real-life research setting of an experienced student facilitator (Figure 3).

This study has its limitations. Firstly, the number of PhD students working with bioinformatics projects in the university is small; possibly limiting the scope of examples to be presented. However, all topics covered in the module were represented by the PhD student presentations. Secondly, it is possible that the students who were selected and subsequently agreed to act as peer-facilitators were unusually engaged. Also, the selection of students was considered essential to maximise the benefits for the peer-receivers, since the development of the peer-facilitators’ bioinformatics and employability skills was not the only objective. It remains to be seen if the outcome would be similar if student selection by staff did not occur.

The positive outcome of this study has led to the incorporation of the activity herein described in the formal module structure. The constant requests from peer-receivers to act as peer-facilitators in following years ensure the continuation of the activity, while emphasising their appreciation of the module dynamic. The intervention was implemented in 2013–14 academic year and it has now run for four consecutive years. In addition, the same intervention has now been incorporated into an undergraduate bioinformatics module at the same university; and into a national Protein Modelling course (sponsored by the Biochemical Society). Importantly, this approach is not limited to bioinformatics, and has also been exploited in different contexts, such as research internships. The use of the same approach in a statistics module is currently under discussion. We are looking for other opportunities to involve a larger number of students.

In conclusion, the increasing importance of teaching experience in determining the likelihood of employment in popular career paths following postgraduate study contrasts sharply with the lack of such experience provided throughout standard PhD training. Similarly, peer-assisted learning, also a key transferrable skill, is not a concept that typically appears on the average PhD training syllabus. Here we claim that we have successfully utilised an inter-cohort peer-assisted learning and student–staff academic partnership; with the effect of improving the students’ understanding of the applicability of cutting-edge bioinformatics techniques, as well as increasing engagement and participation. This study should provide a basis for improving the career prospects of postgraduate students by filling in the gap in training that is evident during the typical PhD course. The plethora of skills and experiences that are developed as a result of this study, in addition to teaching and peer-assisted learning, includes many generally valued by employers – such as autonomy; communication; time-management; teamwork and leadership. The impact on career prospects is therefore not exclusive to education-focussed employment sectors. One peer-facilitator has graduated since their involvement in the study, and has since been able to obtain a position as a post-doctoral research assistant. The peer-facilitator provided feedback that suggests evidence of the propensity for this kind of study to enhance employment prospects across a range of career sectors. Validation of these effects would, however, require significant further study.

## Disclosure statement

No potential conflict of interest was reported by the author.

## Funding

This work was supported by the MRC – Arthritis Research UK Centre for Integrated research into Musculoskeletal Ageing (CIMA) [grant number MR/K006312/1]; BBSRC [grant number BB/K501189/1].
